# Genomic Evidence for Sequestration of Influenza A Virus Lineages in Sea Duck Host Species

**DOI:** 10.3390/v13020172

**Published:** 2021-01-24

**Authors:** Dillon S. McBride, Sarah E. Lauterbach, Yao-Tsun Li, Gavin J. D. Smith, Mary Lea Killian, Jacqueline M. Nolting, Yvonne C. F. Su, Andrew S. Bowman

**Affiliations:** 1 Department of Veterinary Preventive Medicine, College of Veterinary Medicine, The Ohio State University, Columbus, OH 43210, USA; mcbride.338@osu.edu (D.S.M.); lauterbach.7@osu.edu (S.E.L.); nolting.4@osu.edu (J.M.N.); 2 Programme in Emerging Infectious Diseases, Duke-NUS Medical School, Singapore 169857, Singapore; yaotli@u.duke.nus.edu (Y.-T.L.); gavin.smith@duke-nus.edu.sg (G.J.D.S.); yvonne.su@duke-nus.edu.sg (Y.C.F.S.); 3 Diagnostic Virology Laboratory, National Veterinary Services Laboratories, APHIS, USDA, 1920 Dayton Avenue, Ames, IA 50010, USA; mary.l.killian@usda.gov

**Keywords:** influenza A virus, ducks, birds, genomics, prevalence, United States, Great Lakes region

## Abstract

Wild birds are considered the natural reservoir of influenza A viruses (IAVs) making them critical for IAV surveillance efforts. While sea ducks have played a role in novel IAV emergence events that threatened food security and public health, very few surveillance samples have been collected from sea duck hosts. From 2014–2018, we conducted surveillance focused in the Mississippi flyway, USA at locations where sea duck harvest has been relatively successful compared to our other sampling locations. Our surveillance yielded 1662 samples from sea ducks, from which we recovered 77 IAV isolates. Our analyses identified persistence of sea duck specific IAV lineages across multiple years. We also recovered sea duck origin IAVs containing an H4 gene highly divergent from the majority of North American H4-HA with clade node age of over 65 years. Identification of IAVs with long branch lengths is indicative of substantial genomic change consistent with persistence without detection by surveillance efforts. Sea ducks play a role in the movement and long-term persistence of IAVs and are likely harboring more undetected IAV diversity. Sea ducks should be a point of emphasis for future North American wild bird IAV surveillance efforts.

## 1. Introduction

Wild birds including waterfowl, shorebirds, and gulls are recognized as the natural reservoir for influenza A viruses (IAVs) [1]. Because many populations of wild waterfowl migrate, they have been implicated in long distance dispersal and inter-hemispheric movement of IAV [2,3]. Likely a result of this mobility coupled with high natural IAV diversity, wild bird populations have contributed to movement and emergence of economically devastating high pathogenic avian influenza (HPAI) outbreaks [4,5,6]. In addition to agricultural impact, avian-origin IAVs have contributed directly or indirectly to all of the modern influenza pandemics [7,8,9]. IAV reassortment and interspecies transmission events are a primary concern for mitigation of pandemic potential IAV emergence, making IAV diversity circulating in avian hosts an acute threat to public health [10].

Due to the implications of avian-origin IAV, wild bird populations, especially waterfowl, have historically been a focus for influenza surveillance efforts. However, waterfowl species are surveilled at different intensities [11]. Because some subtypes are recovered more frequently from certain species of wild bird [12], it can create a bias that makes certain subtypes appear rarer than others. In 2010, H14-HA was isolated in North American sea ducks in Wisconsin, which was the first detection since its initial description in 1982 near the Caspian Sea [13]. Similarly, several Eurasian-lineage IAV gene segments were isolated from sea ducks in the Midwestern United States during 2010–2011 [14]. Sea ducks in the United States were also associated with the emergence of the turkey HPAI outbreak in 2016 [6]. The emergence of these viruses in the Mississippi flyway was unexpected due to the limited IAV surveillance that had been conducted in sea duck hosts at the time. The lack of surveillance in sea ducks allowed anomalous IAV lineages to remain unsampled for long durations prior to reemergence with substantial genomic change. Very few IAV samples had been previously collected from sea ducks, which are a subset of diving ducks. Prior to 2014, only 64 out of 7865 IAV strains in the USA from avian hosts in the Influenza Research Database (IRD) were isolated from sea duck host species. The dearth of IAVs from sea ducks appears to be a function of under-surveilling of sea ducks. A study that aggregated North American avian IAV surveillance data from 1976–2015 indicated that <1% (745/77,969) of surveillance samples were collected from sea ducks [11]. Another study found that sea ducks have relatively high seroprevalence (61%) with low frequency of virus recovery (0.4%) compared to dabbling ducks indicating current surveillance efforts may fail to capture sea duck IAV infections [15].

Samples are more difficult to collect from sea ducks compared to dabbling ducks, especially when investigators rely upon hunter harvested birds for IAV surveillance. Sea ducks, a subgroup of diving ducks, are classified in the taxonomic tribe Mergini within the family Anatidae and most species make use of saltwater habitat for at least part of the year [16]. While sea duck ecology and habitat are poorly understood relative to other waterfowl, prior work indicates sea ducks breed at very high latitudes, and when they winter in the Mississippi flyway, they make use of offshore habitat in the Great Lakes [17]. Sea duck telemetry data confirm multispecies use and congregation at staging sites as well as movement between nonbreeding sites [18]. However, limited data suggest that wintering sea ducks at Lake Michigan may be the exception, with strong isolation from the other wintering sites in the study [18]. Their winter migration and use of offshore habitat complicates harvest for hunters in the Great Lakes, therefore limiting the locations and opportunities for Mississippi flyway IAV surveillance in these species. These differences in sea duck ecology, migratory behavior, and unique habitat use may result in unique influenza dynamics compared to the more heavily sampled dabbling ducks. We hypothesized that IAVs isolated from sea duck host species would have unique evolutionary lineages due to ecological isolation from more commonly surveyed waterfowl. From 2014–2018, we conducted surveillance focused at locations where sea duck hunting is relatively successful to increase the number of IAV sequences from sea duck hosts and used phylogenetic analyses to investigate clustering of sea duck IAV lineages.

## 2. Materials and Methods

North American sea duck species considered include Barrow’s goldeneye (*Bucephala islandica*), black scoter (*Melanitta nigra*), bufflehead (*Bucephala albeola*), common eider (*Somateria mollissima*), common goldeneye (*Bucephala clangula*), common merganser (*Mergus merganser*), harlequin duck (*Histrionicus histrionicus*), hooded merganser (*Lophodytes cucullatus*), king eider (*Somateria spectabilis*), long-tailed duck (*Clangula hyemalis*), red-breasted merganser (*Mergus serrator*), spectacled eider (*Somateria fischeri*), Steller’s eider (*Polysticta stelleri*), surf scoter *(Melanitta perspicillata*), and white-winged scoter (*Melanitta fusca*) [19].

Cloacal swabs were collected from hunter-harvested birds from 2014 through 2018 as part of a multi-year active IAV surveillance project, under United States Fish and Wildlife Services Scientific Collection Permit number MB66162B-0 and the Ohio State University Institutional Animal Care and Use Committee approved protocol number 2007A0148. The Mississippi flyway, where anomalous sea duck emergent events have been previously described [6,13,14], was the target for the majority of our surveillance. Sampling efforts were focused at locations where sea duck harvest is successful, especially Green Bay, WI, USA. Samples were subjected to virus isolation in embryonated chicken eggs as previously described [20]. Statistics and prevalence were estimated using STATA Statistical Software release 14. IAV isolates were sent to the National Veterinary Services Laboratories (Ames, IA, USA) for full genome sequencing. Following reverse transcription, cDNA libraries were prepared and sequencing using the Illumina MiSeq platform [21]. Complete sequences were used to determine HA-NA subtypes. All sequences generated by this study are publicly available in GenBank, accession numbers are available in Supplementary Table S1. In order to appropriately investigate patterns and abnormalities of IAVs isolated from sea duck host species, all additional IAV sequences from 2018 or earlier from avian host species were downloaded from the IRD [22].

Nucleotide sequences were aligned using MAFFT v7.409 [23]. Alignments were manually examined and restricted to coding regions. Phylogenies were estimated for each gene segment individually using FastTree in order to infer basic topology and relatedness [24]. Clades containing sea duck IAVs were identified, and data were subsampled for further analysis. Maximum likelihood (ML) phylogenetic analyses were completed using RAxML v8.2.10 with a general time reversable (GTR) plus gamma substitution model and a maximum 1000 bootstrap replicates [25]. ML trees were midpoint rooted for clarity. Time-scaled phylogenies and time to most recent common ancestor (TMRCA) were estimated using Bayesian Markov Chain Monte Carlo analysis implemented in BEAST v1.10.4 [26]. We evaluated models using jModelTest2 [27] and conducted further model comparison using the path sampling method of marginal likelihood estimations implemented in BEAST [28,29]. We selected a GTR plus gamma substitution model with an uncorrelated lognormal relaxed clock [30]. Two independent runs were completed with chain length of 50 million generations and combined for analysis. The outputs were visualized with Tracer v1.7.1 to ensure proper burn-in and effective sample sizes >200 for relevant parameters. The maximum clade credibility (MCC) tree was summarized with TreeAnnotator v1.10.4. All trees were visualized and edited using FigTree v1.4.3 and Adobe Illustrator.

## 3. Results

### 3.1. IAV Surveillance

From 2014–2018, we collected a total of 1662 cloacal swab samples from nine sea duck species across eight states (Table 1). Based on virus isolation results, estimated prevalence of IAV in sea ducks was 4.63% (77/1662) (95% CI 3.67–5.76%). We recovered 80.5% of sea duck IAV isolates from samples collected in Wisconsin, of which 85.5% were from the two most frequently sampled species in this study; common goldeneyes and long-tailed ducks. (Table 1). From birds sampled in Wisconsin, the estimated prevalence of IAV in common goldeneyes was 7.3% (45/616, 95% CI 5.38–9.65%) compared to 1.6% in long-tailed ducks (8/485, 95% CI 0.715–3.22%). Common goldeneyes and long-tailed ducks had similar sample sizes at the same sampling locations (Table 1), yet we estimated significantly higher prevalence in common goldeneyes compared to long-tailed ducks. We sequenced 74 of our 77 viral isolates revealing 24 different HA-NA subtype combinations, with H4N8 accounting for over 40% of sequenced subtypes (Table 2).

### 3.2. Phylogenetic Analyses

**Figure 1:**
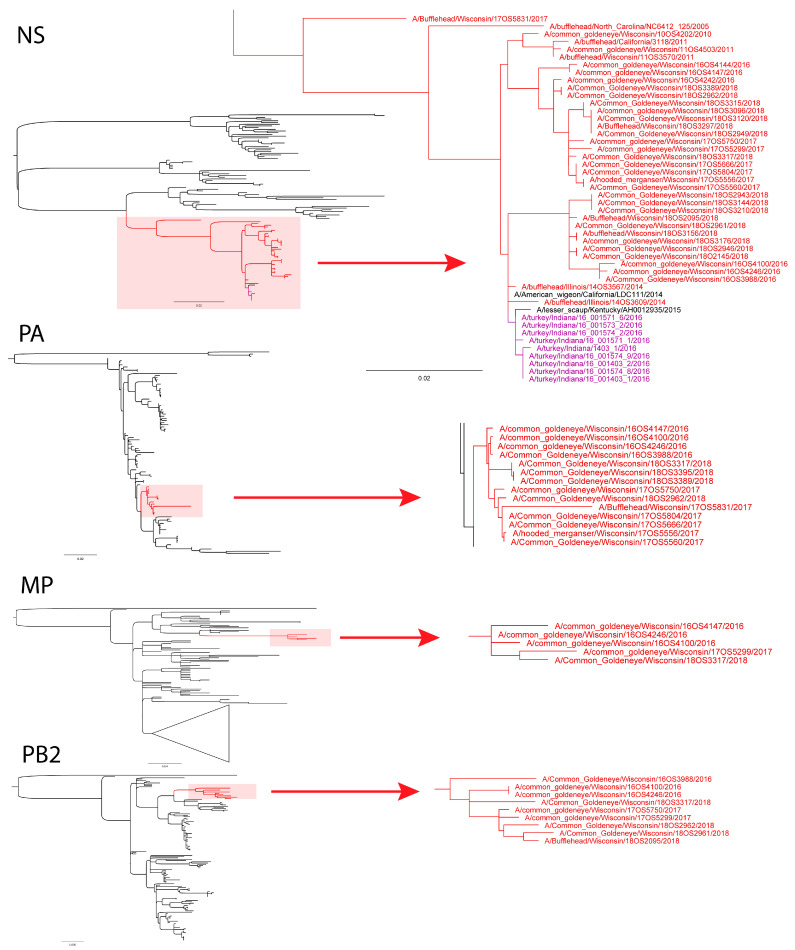
Subsampled clade maximum likelihood phylogenies showing uninterrupted persistence of influenza A virus (IAV) lineages in the NS, PA, MP, and PB2 gene segments. Clustering of sea duck origin viruses from 2016–2018 indicate that surveillance efforts did not detect these lineages in other species or locations during the study timeframe. Sea duck IAVs are shown in red and 2016 Indiana HPAI turkey outbreak IAVs are shown in purple. NEXUS tree files can be found in Supplemental files S1–S4.

**Figure 2:**
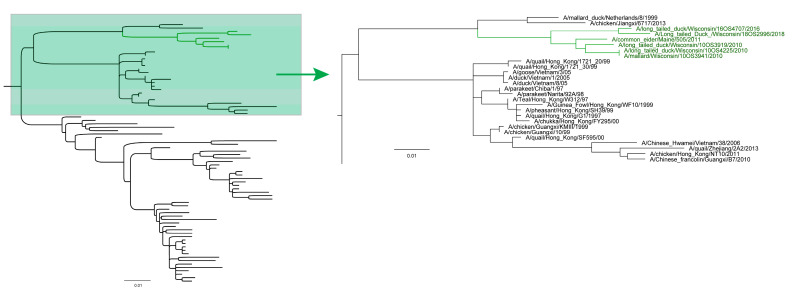
Subsampled maximum likelihood tree estimated from the NS segment analysis showing a Eurasian lineage sea duck clade (green) and the later recovered 2016 and 2018 IAV with long branch length. Eurasian origin NS segments have persisted in sea ducks, and the long branch leading to 16OS4707 and 18OS2996 indicates that the clade may have diversified earlier than the initial detection in 2010 indicated. NEXUS tree file can be found in supplementary file 5.

We found consistent clustering of sea duck origin IAVs in four internal gene segments NS, PA, MP, and PB2 (Figure 1). Monophyly of sea duck IAV sequences from multiple years is indicative of host specific isolation of viruses within these clades. IAVs containing one or more gene segments belonging to persistent clades were recovered from sea ducks during 2016–2018 in Wisconsin. In the case of the NS segment (Figure 1, Supplemental file S1), the clade also includes earlier sea duck origin IAVs from Wisconsin in 2010 and 2011 and encompasses NS segments with >98% sequence identity and close phylogenetic relationship to those in the 2016 Indiana turkey HPAI outbreak [6]. In many cases, more than one segment was detected within a persistent clade including 3 strains containing all four persistent gene segments, 5 strains containing 3/4, and 7 strains containing 2/4 (Table 3). Eurasian-origin divergent NS sequences were isolated from A/long-tailed duck/Wisconsin/16OS4707/2016 and A/long-tailed duck/Wisconsin/18OS2996/2018 which share a most recent common ancestor (Figure 2, Supplemental file S5). This NS lineage has been detected in Wisconsin long-tailed ducks in 2011, 2016, and 2018 providing supporting evidence that isolation of sea duck hosts may allow for persistence of IAV diversity through time in one species and location.

**Figure 3:**
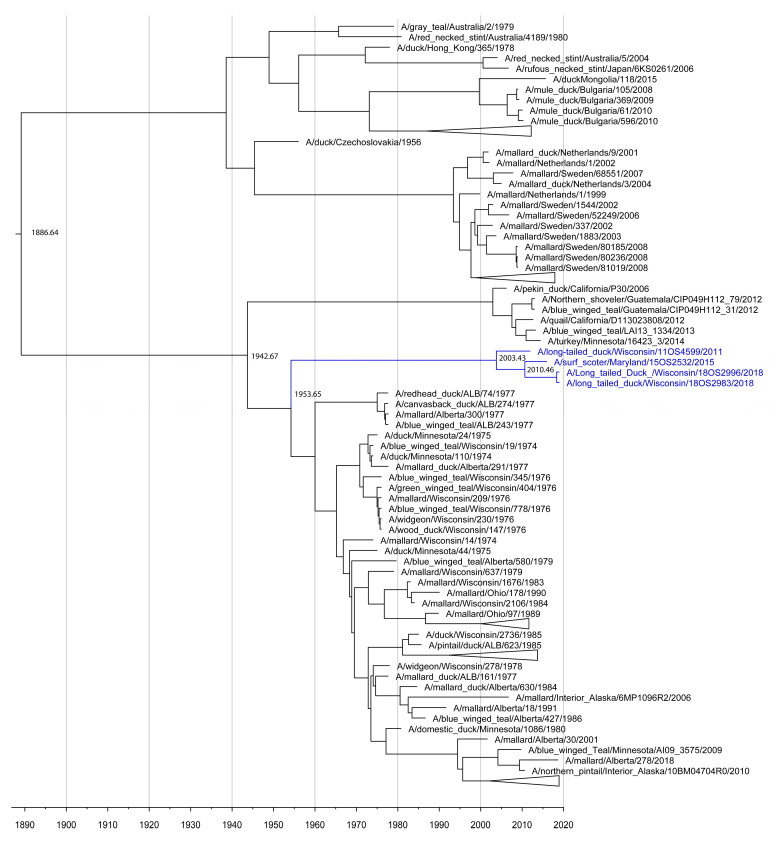
The maximum clade credibility (MCC) tree estimated from the BEAST H4 segment analysis indicates 2011, 2015, and 2018 sea duck IAVs (blue) as sister taxa to the majority of the remaining North American lineage H4 viruses with very long time to most recent common ancestor for the monophyletic clade. This clade also contains relatively long internal branch lengths and detection in both Wisconsin and Maryland. Dates are indicated on nodes and time scale.

A/long-tailed duck/Wisconsin/18OS2996/2018 also contained an H4-HA segment from a deeply divergent sea duck-origin clade in the H4 phylogeny, a sister group to the majority of North American H4 clade (Figure 3, Table 3). The TMRCA of the sea duck clade is 65.26 years (age 1942.53–1964.46 95% highest posterior density (HPD), posterior probability 99.7%) and is only 2 descendant nodes from the primary North American-Eurasian H4 split. This clade contained a total of 4 sea duck origin IAVs—A/long-tailed duck/Wisconsin/18OS2996/201, A/long-tailed duck/Wisconsin/18OS2983/2018, A/surf scoter/Maryland/15OS2532/2015, and A/long-tailed duck/Wisconsin/11OS4599/2011. The long branch and exclusive isolation from sea duck hosts may reflect long term persistence in sea ducks without detection in avian hosts due to under sampling. The M segment of A/long-tailed duck/Wisconsin/11OS4599/2011 clusters in a clade containing 2010 H14 IAVs with relatively short branch lengths. The basal node (age 2009.35–2010.78 95% HPD) for that M clade has 100% posterior probability, but there is high uncertainty within the clade likely due to the close relation.

## 4. Discussion

We found strong evidence that specific IAV lineages are sequestered in sea duck host species. Out of the 74 IAVs sequenced, 35 had at least one gene segment that clustered in a monophyletic clade of sea duck origin viruses that were detected across several years (Table 3). Persistence of IAVs in one species and location throughout multiple seasons has also been described in mallard ducks in the Mississippi flyway [31]. The large sampling bias in favor of dabbling ducks can make any trait inferences regarding sea duck host specificity difficult to evaluate. However, the monophyly and branch length of these IAVs recovered from exclusively sea duck samples provides a compelling case that there is not frequent gene flow of IAV from sea ducks into other waterfowl host systems. We did not measure host factors of sea ducks, so we cannot make specific conclusions regarding mechanisms of IAV host-specificity. While still poorly understood, sea ducks display complex habitat use, ecology, and migration patterns that differs greatly from dabbling ducks [17,18]. The sharing of habitat and breeding grounds likely plays a role in interspecific transmission and transcontinental movement of IAV [32]. Because abiotic factors and environmental transmission can play a role in persistence of avian IAV outbreaks [33,34,35,36], the unique habitat use and ecological niche of sea ducks likely plays a role in the host specific isolation of IAV lineages.

The detection of a persistent sea duck lineage NS segment in such close relation to recent HPAI viruses from US poultry is of critical relevance. Evaluation of recent IAV viruses from sea ducks showed five gene segments with >98% similarity to the Indiana turkey HPAI H7N8 outbreak in 2016, indicating that sea ducks are an important niche of viral diversity when it comes to risk of introduction to poultry [6]. IAV from sea duck hosts have contributed to a major agricultural event at least once and given the natural diversity of IAV in wild birds, have the potential to contribute to spillover events and emerging zoonosis in the future. Zoonotic transmission of IAV from poultry remains relevant as illustrated by the pandemic potential of the avian origin H7N9 IAV in China [37]. We also sequenced three IAVs with multiple HA-NA subtypes. These mixed subtype isolates provide evidence for coinfection with multiple IAVs in these hosts. Mixed infections can facilitate viral reassortment, which is an additional pathway for novel virus emergence.

IAV containing a Eurasian lineage NS gene segment was previously described in North American sea ducks in 2009–2010 along with H10-HA and N6-NA gene segments [14]. Analysis of those NS sequences revealed a close relationship to both 2010 North American and 1982 Eurasian H14 IAVs [14]. Repeated detection of this NS clade in North American sea ducks is evidence that the lineage has been circulating undetected in sea ducks since its initial emergence. The branch length in the Eurasian sea duck origin NS clade along with the amount of genomic change in the novel H14 IAV detections in 2010 [2] provides support for the assessment that unique IAVs are circulating undetected in these under-sampled hosts. The exceptionally long branch of the H4 clade leaves it susceptible to long branch effects drawing it closer to the base of the North American clade. However, the length of the branch in and of itself is evidence that the relative under sampling of sea duck host species can result in lineages of IAV remaining undetected for, in this case, a period estimated to be over 60 years. The relationship of the 2010 reemergent H14 IAVs in North America [13] to the A/long-tailed duck/Wisconsin/11OS4599/2011 M segment and the persistent Eurasian lineage NS segments from 2010–2018 is strong evidence of reassortant and diverse IAVs persisting in sea duck host species. Previously unsampled but persistent IAV lineages can rapidly disseminate to new host groups and regions. Shortly after the reemergence of H14 IAV in Mississippi flyway sea ducks [13], surveillance efforts detected additional H14 in North American ducks [14]. H14 viruses were persisting undetected, but shortly after reemergence in sea ducks, H14 was introduced and spread rapidly in dabbling ducks and new locations [38]. This presents a pathway for unsampled or rare IAV diversity to persist undetected in sea ducks and then rapidly disseminate throughout North America upon introduction into new hosts, especially if the new hosts are naïve to that diversity and have more extensive migratory networks and commingling points.

Despite similar sample sizes across all study years, in Wisconsin we isolated IAV at a significantly higher prevalence in common goldeneyes compared to long-tailed ducks. There may be distinct differences in viral dynamics between individual sea duck host species. The estimated IAV prevalence in common goldeneyes is not drastically different than the estimated autumn IAV prevalence described in mallard ducks previously [31], whereas IAV was recovered much less frequently from long-tailed ducks. Not enough about sea duck habitat use is known to determine whether goldeneye ecology more closely reflects that of dabbling ducks compared to long-tailed ducks. We did, however, estimate a much higher IAV prevalence in sea ducks compared to a previous study in the North Atlantic [15]. The majority of samples from this study were collected from the Great Lakes region in the Mississippi flyway, which likely reflects a distinct population of ducks with substantially different ecology and environmental factors compared to the coastal Atlantic flyway.

Gaps in IAV surveillance due to restrictions placed by sampling techniques are well documented. In 2013, an evolutionarily distinct H11N2 IAV lineage was detected in Adélie penguins in Antarctica, with gene segments TMRCA estimates of 49–80 years [39]. Then, in 2015, a reassortant LPAI H5N5 IAV was recovered from a chinstrap penguin with a North American H5 and a Eurasian N5 [40]. These findings highlighted the importance of the previously unsurveyed Antarctic in IAV efforts. Likewise, in North America, the seasonality and timing of bird migration is a critical factor that impacts the number and diversity of IAVs detected in wild birds [41]. Due to the sampling scheme currently in place, sea duck species are often only sampled, at least in the Mississippi flyway, during specific winter months and in low numbers. Many sea duck species breed at very high latitudes and even artic regions, and winter farther south where they are more easily sampled. The brief snapshot in time and migration that samples are collected to survey IAVs in sea duck species is not able to provide the entire picture as to what role they may play in unique diversity, reassortment, and transcontinental movement of IAVs. These new data support the call for diversification of waterfowl species in IAV surveillance. The analyses show substantial genetic change along the branches of sea duck origin IAVs in close relation to viruses associated with significant emergent events in the United States. Sea ducks should not be ignored as significant disseminators of unique and diverse IAVs and contributors to outbreak events relevant to both agriculture and public health. Although sea ducks are difficult to sample, especially throughout the year, they should be targets for IAV surveillance efforts.

## Figures and Tables

**Table 1:** Number of sea duck samples (number of influenza A virus isolates) collected from 2014–2018 by state and species.

Species	State									Total
	DE	IL	MD	MI	MO	MS	OH	TN	WI	
***Black Scoter***			45(6)						1	46(6)
***Bufflehead***	6	21(3)	44	3		2	12	6	64(8)	158(11)
***Common goldeneye***				8(1)			5		616(45)	629(46)
***Common merganser***		1	1	1			2		20	25
***Hooded merganser***	5	10	5	2	2	14	14	1	20	73
***Long-tailed duck***			42	60					485(8)	587(8)
***Red breasted merganser***				3			1		12(1)	16(1)
***Surf scoter***	1		92(5)						2	95(5)
***White-winged scoter***			10						23	33
***Total***	12	32(3)	239(11)	77(1)	2	16	34	7	1243(62)	1662(77)

**Table 2:** Subtypes of 74 influenza A virus sequences recovered from sea ducks from 2014–2018.

HA Subtype	NA Subtype												Total
	1	2	3	4	5	6	7	8	9	Mixed (4,6)	Mixed (3,8)	Mixed (7,8)	
***1***	1		1										2
***2***	1		2										3
***3***	2					1		1					4
***4***		2				2		30					34
***6***	2												2
***7***	2		3	2		1							8
***10***	1		2			2	5					1	11
***11***									6				6
***12***					2								2
***Mixed (4,11)***											1		1
***Mixed (4,10)***										1			1
***Total***	9	2	8	2	2	6	5	31	6	1	1	1	74

**Table 3:** Sea duck strain clade summarization for the 35 strains with gene segments detected in persistent, sea duck specific lineages. Red indicates lineages which are displayed in Figure 1, with an NS segment highly related to 2016 HPAI turkey viruses. The Eurasian origin NS lineage is shown in green (Figure 2). The deeply divergent H4 clade is shown in blue (Figure 3).

Strain	PB2	PA	HA	MP	NS
A/Bufflehead/Wisconsin/17OS5831/2017					
A/Bufflehead/Wisconsin/18OS2095/2018					
A/Bufflehead/Wisconsin/18OS3156/2018					
A/Bufflehead/Wisconsin/18OS3297/2018					
A/Common goldeneye/Wisconsin/18OS3395/2018					
A/Common goldeneye/Wisconsin/16OS3988/2016					
A/Common goldeneye/Wisconsin/16OS4100/2016					
A/Common goldeneye/Wisconsin/16OS4144/2016					
A/Common goldeneye/Wisconsin/16OS4147/2016					
A/Common goldeneye/Wisconsin/16OS4242/2016					
A/Common goldeneye/Wisconsin/16OS4246/2016					
A/Common goldeneye/Wisconsin/17OS5299/2017					
A/Common goldeneye/Wisconsin/17OS5560/2017					
A/Common goldeneye/Wisconsin/17OS5666/2017					
A/Common goldeneye/Wisconsin/17OS5750/2017					
A/Common goldeneye/Wisconsin/17OS5804/2017					
A/Common goldeneye/Wisconsin/18O2145/2018					
A/Common goldeneye/Wisconsin/18OS2943/2018					
A/Common goldeneye/Wisconsin/18OS2946/2018					
A/Common goldeneye/Wisconsin/18OS2949/2018					
A/Common goldeneye/Wisconsin/18OS2961/2018					
A/Common goldeneye/Wisconsin/18OS2962/2018					
A/Common goldeneye/Wisconsin/18OS3096/2018					
A/Common goldeneye/Wisconsin/18OS3120/2018					
A/Common goldeneye/Wisconsin/18OS3144/2018					
A/Common goldeneye/Wisconsin/18OS3176/2018					
A/Common goldeneye/Wisconsin/18OS3210/2018					
A/Common goldeneye/Wisconsin/18OS3315/2018					
A/Common goldeneye/Wisconsin/18OS3317/2018					
A/Common goldeneye/Wisconsin/18OS3389/2018					
A/Hooded merganser/Wisconsin/17OS5556/2017					
A/Long-tailed duck/Wisconsin/16OS4707/2016					
A/Long-tailed duck/Wisconsin/18OS2983/2018					
A/Long-tailed duck/Wisconsin/18OS2996/2018					
A/Surf scoter/Maryland/15OS2532/2015					

## Data Availability

All the IAV sequences generated as a part of this study are publicly available in GenBank, individual accession numbers are provided in Supplemental Table S1.

## Supplementary Materials

The following are available online at https://www.mdpi.com/1999-4915/13/2/172/s1, Table S1: GenBank accession numbers for strains sequenced as a part of this study. Supplemental files S1–S4: NEXUS tree files showing the full subsampled clades displayed in Figure 1. Supplemental file S5: NEXUS tree file showing the full subsampled clade displayed in Figure 2.
