# Supplementary material for: Genomic Evidence for Sequestration of Influenza A Virus Lineages in Sea Duck Host Species
Source: Viruses. 2021 Jan 24;13(2):172. doi: 10.3390/v13020172 (PMC7911388; doi:10.3390/v13020172)
Supplement: Supplementary file 1 [file viruses-13-00172-s001.zip › supplemental table 1.docx]

**Table S1**. GenBank accession numbers for strains sequenced as a part of this study

| Strain Name | 1 PB2 | 2 PB1 | 3 PA | 4 HA | 5 NP | 6 NA | 7 MP | 8 NS |
| --- | --- | --- | --- | --- | --- | --- | --- | --- |
| A/black scoter/Maryland/15OS2527/2015 | MT829164 | MT829168 | MT829163 | MT829162 | MT829165 | MT829167 | MT829169 | MT829166 |
| A/bufflehead/Illinois/14OS3567/2014 | KY463955 | KY463916 | KY463949 | -N/A- | KY463963 | KY463936 | KY463891 | KY463849 |
| A/bufflehead/Illinois/14OS3609/2014 | KY463845 | KY463878 | KY463841 | -N/A- | KY463877 | KY463907 | KY463922 | KY463795 |
| A/bufflehead/Wisconsin/17OS4051/2017 | MK237300 | MK236756 | MK237772 | MK237598 | MK237447 | MK237740 | MK236650 | MK236661 |
| A/bufflehead/Wisconsin/17OS5636/2017 | MK237595 | MK236831 | MK236706 | MK237891 | MK237815 | MK237140 | MK237710 | MK237558 |
| A/Bufflehead/Wisconsin/17OS5786/2017 | MT824687 | MT824684 | MT824683 | MT824685 | MT824686 | MT824682 | MT824689 | MT824688 |
| A/bufflehead/Wisconsin/17OS5788/2017 | MK237221 | MK237262 | MK236826 | MK237202 | MK237519 | MK237720 | MK237105 | MK237807 |
| A/Bufflehead/Wisconsin/17OS5831/2017 | MT824718 | MT824714 | MT825033 | MT824717 | MT824716 | MT824719 | MT824715 | MT824720 |
| A/Bufflehead/Wisconsin/18OS2095/2018 | MN430966 | MN430970 | MN430969 | MN430968 | MN430967 | MN430971 | MN430964 | MN430965 |
| A/bufflehead/Wisconsin/18OS3156/2018 | MN795023 | MN795024 | MN795025 | MN795026 | MN795029 | MN795022 | MN795027 | MN795028 |
| A/Bufflehead/Wisconsin/18OS3297/2018 | MN430788 | MN430793 | MN430789 | MN430790 | MN430794 | MN430792 | MN430791 | MN430795 |
| A/common goldeneye/Michigan/18OS3858/2018 | MN795014 | MN795020 | MN795015 | MN795016 | MN795019 | MN795017 | MN795021 | MN795018 |
| A/Common Goldeneye/Wisconsin/16OS3988/2016 | MN552510 | MN552508 | MN552513 | MN552512 | MN552506 | MN552511 | MN552509 | MN552507 |
| A/common goldeneye/Wisconsin/16OS4100/2016 | MG280253 | MG280264 | MG280572 | MG280162 | MG279822 | MG280295 | MG280581 | MG280028 |
| A/common goldeneye/Wisconsin/16OS4144/2016 | MG279784 | MG280559 | MG280474 | MG280065 | MG280349 | MG279775 | MG279835 | MG279932 |
| A/common goldeneye/Wisconsin/16OS4147/2016 | MG279874 | MG280078 | MG280222 | MG280191 | MG280531 | MG280035 | MG280231 | MG280511 |
| A/common goldeneye/Wisconsin/16OS4242/2016 | MG280240 | MG280502 | MG280421 | MG280158 | MG280167 | MG280594 | MG280341 | MG280328 |
| A/common goldeneye/Wisconsin/16OS4246/2016 | MG280220 | MG280081 | MG280276 | MG280169 | MG280213 | MG279766 | MG280294 | MG280352 |
| A/common goldeneye/Wisconsin/17OS4053/2017 | MK236951  MT824883 | MK237836  MT825002 | MK237288  MT824885 | MK237042  MT824886 | MK237811  MT824887 | MK237576  MT824884 | MK237283  MT824882 | MK237621  MT824888 |
| A/Common Goldeneye/Wisconsin/17OS4063/2017 | MT824894 | MT824889 | MT824891 | MT824895 | MT824896 | MT824890 | MT824893 | MT824892 |
| A/common goldeneye/Wisconsin/17OS4109/2017 | MK237303 | MK236740 | MK236720 | MK237964 | MK237981 | MK237124 | MK237321 | MK236841 |
| A/Common Goldeneye/Wisconsin/17OS4111/2017 | MT824834 | MT824833 | MT824829 | MT825030  MT824832 | -N/A- | MT825029  MT824831 | MT824830 | MT824828 |
| A/common goldeneye/Wisconsin/17OS5294/2017 | MK237428 | MK237497 | MK237987 | MK236847 | MK237512 | MK237639 | MK237362 | MK237714 |
| A/common goldeneye/Wisconsin/17OS5297/2017 | MK236967 | MK237919 | MK237539 | MK236948 | MK237709 | MK237156 | MK236717 | MK237955 |
| A/common goldeneye/Wisconsin/17OS5299/2017 | MK237932 | MK237126 | MK236958 | MK237743 | MK236686 | MK237365 | MK236998 | MK237633 |
| A/Common Goldeneye/Wisconsin/17OS5307/2017 | MT824864 | MT824859 | MT824862 | MT824866 | MT824865 | MT824860 | MT824861 | MT824863 |
| A/common goldeneye/Wisconsin/17OS5312/2017 | MK236963 | MK236902 | MK236620 | MK237034 | MK237098 | MK237352 | MK237804 | MK237498 |
| A/Common Goldeneye/Wisconsin/17OS5313/2017 | MT824813 | MT824814 | MT824818 | MT824815 | MT824812 | MT824819 | MT824816 | MT824817 |
| A/Common Goldeneye/Wisconsin/17OS5487/2017 | MT824610 | MT824607 | MT824612 | MT824605 | MT824608 | MT824606 | MT824609 | MT824611 |
| A/common goldeneye/Wisconsin/17OS5553/2017 | MK237617 | MK237905 | MK237116 | MK237111 | MK237592 | MK236974 | MK236895 | MK237100 |
| A/Common Goldeneye/Wisconsin/17OS5560/2017 | MT824691 | MT824697 | MT824694 | MT824693 | MT824692 | MT824695 | MT824696 | MT824690 |
| A/Common Goldeneye/Wisconsin/17OS5666/2017 | MT824636 | MT824635 | MT824639 | MT824637 | MT824642 | MT824640 | MT824638 | MT824641 |
| A/common goldeneye/Wisconsin/17OS5750/2017 | MK237323 | MK236775 | MK236790 | MK236924 | MK237347 | MK237007 | MK237863 | MK237260 |
| A/common goldeneye/Wisconsin/17OS5794/2017 | MK237320 | MK236739 | MK237110 | MK236746 | MK236788 | MK236787 | MK237775 | MK237580 |
| A/Common Goldeneye/Wisconsin/17OS5804/2017 | MT824630 | MT824632 | MT824627 | MT824634 | MT824628 | MT824629 | MT824631 | MT824633 |
| A/common goldeneye/Wisconsin/17OS5813/2017 | MK236866 | MK237841 | MK236636 | MK237624 | MK236623 | MK237290 | MK237622 | MK237641 |
| A/common goldeneye/Wisconsin/17OS5819/2017 | MK236773 | MK236753 | MK236613 | MK236762 | MK236876 | MK237360 | MK237261 | MK237591 |
| A/common goldeneye/Wisconsin/17OS5833/2017 | MK237233 | MK236815 | MK237707 | MK237191 | MK237087 | MK237165 | MK237941 | MK236947 |
| A/Common Goldeneye/Wisconsin/18O2145/2018 | MN430983 | MN430982 | MN430986 | MN430985 | MN430987 | MN430980 | MN430984 | MN430981 |
| A/Common Goldeneye/Wisconsin/18OS2943/2018 | MN430828 | MN430829 | MN430830 | MN430835 | MN430833 | MN430831 | MN430832 | MN430834 |
| A/Common Goldeneye/Wisconsin/18OS2946/2018 | MN430823 | MN430826 | MN430820 | MN430821 | MN430825 | MN430824 | MN430827 | MN430822 |
| A/Common Goldeneye/Wisconsin/18OS2949/2018 | MN430855 | MN430853 | MN430856 | MN430852 | MN430854 | MN430858 | MN430857 | MN430859 |
| A/Common Goldeneye/Wisconsin/18OS2951/2018 | MT824761 | MT824754 | MT824760 | MT824755 | MT824758 | MT824757 | MT824759 | MT824756 |
| A/common goldeneye/Wisconsin/18OS2955/2018 | MN794956 | MN794961 | MN794959 | MN794962 | MN794958 | MN794957 | MN794963 | MN794960 |
| A/Common Goldeneye/Wisconsin/18OS2961/2018 | MN430818 | MN430812 | MN430814 | MN430815 | MN430817 | MN430813 | MN430819 | MN430816 |
| A/Common Goldeneye/Wisconsin/18OS2962/2018 | MN430802 | MN430797 | MN430801 | MN430796 | MN430799 | MN430798 | MN430800 | MN430803 |
| A/Common Goldeneye/Wisconsin/18OS3095/2018 | MT825082 | MT825084 | MT825081 | MT825083 | MT825085 | MT825080 | MT825079 | -N/A- |
| A/common goldeneye/Wisconsin/18OS3096/2018 | MN794975 | MN794977 | MN794978 | MN794972 | MN794979 | MN794974 | MN794976 | MN794973 |
| A/Common Goldeneye/Wisconsin/18OS3120/2018 | MT824582 | MT824588 | MT824581 | MT824583 | MT824585 | MT824587 | MT824584 | MT824586 |
| A/Common Goldeneye/Wisconsin/18OS3144/2018 | MT825040 | MT825041 | MT825045 | MT825042 | MT825039 | MT825044  MT825054 | MT825043 | MT825038 |
| A/common goldeneye/Wisconsin/18OS3176/2018 | MN794996 | MN794990 | MN794992 | MN794995 | MN794997 | MN794991 | MN794993 | MN794994 |
| A/Common Goldeneye/Wisconsin/18OS3210/2018 | MN430784 | MN430780 | MN430787 | MN430782 | MN430781 | MN430785 | MN430783 | MN430786 |
| A/Common Goldeneye/Wisconsin/18OS3315/2018 | MN430766 | MN430768 | MN430769 | MN430764 | MN430767 | MN430771 | MN430765 | MN430770 |
| A/Common Goldeneye/Wisconsin/18OS3317/2018 | MN430730 | MN430728 | MN430732 | MN430727 | MN430725 | MN430729 | MN430731 | MN430726 |
| A/Common Goldeneye/Wisconsin/18OS3389/2018 | MN430763 | MN430757 | MN430761 | MN430762 | MN430759 | MN430758 | MN430760 | MN430756 |
| A/Common Goldeneye/Wisconsin/18OS3390/2018 | MT824909 | MT824906 | MT824910 | MT824907 | MT824905 | MT824908 | MT824911 | MT824912 |
| A/Common Goldeneye/Wisconsin/18OS3395/2018 | MT825073 | MT825076 | MT825078 | MT825072 | MT825071 | MT825077 | MT825075 | MT825074 |
| A/hooded merganser/Wisconsin/17OS5556/2017 | MK237316 | MK237500 | MK237886 | MK237829 | MK237880 | MK237411 | MK237518 | MK236820 |
| A/Long-tailed Duck /Wisconsin/18OS2996/2018 | MT824590 | MT824589 | MT825006 | MT825008  MT824593 | MT824592 | MT824591  MT824594 | MT825009 | MT825007 |
| A/long-tailed duck/Wisconsin/16OS4632/2016 | MG280172 | MG279918 | MG280525 | MG280318 | MG280188 | MG280563 | MG279971 | MG280438 |
| A/long-tailed duck/Wisconsin/16OS4707/2016 | MG280192 | MG279961 | MG279892 | MG280542 | MG279826 | MG280364 | MG280124 | MG279792 |
| A/long-tailed duck/Wisconsin/16OS5291/2016 | MG280027 | MG279924 | MG280195 | MG280473 | MG280416 | MG280039 | MG280381 | MG280566 |
| A/long-tailed duck/Wisconsin/18OS2983/2018 | MN794949 | MN794950 | MN794948 | MN794954 | MN794952 | MN794955 | MN794953 | MN794951 |
| A/long-tailed duck/Wisconsin/18OS2993/2018 | MN794965 | MN794971 | MN794968 | MN794969 | MN794967 | MN794970 | MN794964 | MN794966 |
| A/long-tailed duck/Wisconsin/18OS3407/2018 | MN795010 | MN795012 | MN795011 | MN795013 | MN795006 | MN795007 | MN795008 | MN795009 |
| A/long-tailed duck/Wisconsin/18OS3416/2018 | MN795040 | MN795043 | MN795039 | MN795042 | MN795038 | MN795045 | MN795041 | MN795044 |
| A/scoter/Maryland/16OS2525/2016 | MG280463 | MG280359 | MG280137 | MG280406 | MG280561 | MG280095 | MG280134 | MG280183 |
| A/scoter/Maryland/16OS2529/2016 | MG280279 | MG280439 | MG280224 | MG280022 | MG280371 | MG280344 | MG280019 | MG280234 |
| A/scoter/Maryland/16OS2531/2016 | MG280085 | MG280161 | MG280350 | MG279937 | MG280262 | MG280115 | MG280385 | MG280538 |
| A/surf scoter/Maryland/14OS1407/2014 | CY204665 | CY204664 | CY204663 | CY204658 | CY204661 | CY204660 | CY204659 | CY204662 |
| A/surf scoter/Maryland/14OS1418/2014 | CY204673 | CY204672 | CY204671 | CY204666 | CY204669 | CY204668 | CY204667 | CY204670 |
| A/surf scoter/Maryland/15OS2485/2015 | MT829157 | MT829156 | MT829155 | MT829158 | MT829159 | MT829152 | MT829153 | MT829154 |
| A/surf scoter/Maryland/15OS2486/2015 | MT829176 | MT829173 | MT829177 | MT829170 | MT829171 | MT829175 | MT829174 | MT829172 |
| A/surf scoter/Maryland/15OS2532/2015 | MT824927 | MT824925 | MT824923 | MT825037 | MT824926 | MT824921 | MT824924 | MT824922 |
